# Supplementary material for: Long-Term Observational Outcomes after Total Correction of Congenital Heart Disease in Korean Patients with Down Syndrome: A National Cohort Study
Source: Children (Basel). 2022 Aug 31;9(9):1329. doi: 10.3390/children9091329 (PMC9497944; doi:10.3390/children9091329)
Supplement: Supplementary file 1 [file children-09-01329-s001.zip › children-1812650-supplementary.pdf]

children

Table S1 The composition of congenital heart disease by ICD 10 code

CHD diagnosis	ICD10 code	Congenital malformations of the circulatory system(Q20-Q28)
Others	Q20.0	Common arterial trunk
	Q20.2	Double outlet left ventricle
	Q21.8	Other congenital malformations of cardiac septa
	Q21.9	Congenital malformation of cardiac septum, unspecified
	Q22.2	Congenital pulmonary valve insufficiency
	Q22.3	Other congenital malformations of pulmonary valve
	Q22.4	Congenital tricuspid stenosis
	Q22.5	Ebstein anomaly
	Q22.8	Other congenital malformations of tricuspid valve
	Q22.9	Congenital malformation of tricuspid valve, unspecified
	Q23.8	Other congenital malformations of aortic and mitral valves
	Q23.9	Congenital malformation of aortic and mitral valves, unspecified
	Q24.2	Cor triatriatum
	Q24.5	Malformation of coronary vessels
	Q24.8	Other specified congenital malformations of heart
	Q24.9	Congenital malformation of heart, unspecified
	Q25.7	Other congenital malformations of pulmonary artery
	Q25.8	Other congenital malformations of great arteries
	Q25.9	Congenital malformation of great arteries, unspecified
DORV	Q20.1	Double outlet right ventricle
TGA	Q20.3	Discordant ventriculoarterial connection
	Q20.5	Discordant atrioventricular connection
	Q20.8	Other congenital malformations of cardiac chambers and connections
	Q20.9	Congenital malformation of cardiac chambers and connections, unspecified
SV	Q20.4	Double inlet ventricle
	Q22.6	Hypoplastic right heart syndrome
	Q23.4	Hypoplastic left heart syndrome
VSD	Q21	Congenital malformations of cardiac septa
	Q21.0	Ventricular septal defect
	Q21.00	Muscular Ventricular septal defect
	Q21.01	Perimembranous Ventricular septal defect
	Q21.08	Other ventricular septal defect
	Q21.09	Ventricular septal defect, unspecified
ASD	Q21.1	Atrial septal defect
	Q21.10	Patent or persistent foramen ovale

	Q21.11	
	Q21.18	Other atrial septal defect
	Q21.19	Atrial septal defect, unspecified
AVSD	Q21.2	Atrioventricular septal defect
PDA	Q21.4	Aortopulmonary septal defect
	Q25.0	Patent ductus arteriosus
PA	Q22.0	Pulmonary valve atresia
	Q25.5	Atresia of pulmonary artery
PS	Q22.1	Congenital pulmonary valve stenosis
	Q24.3	Pulmonary infundibular stenosis
	Q25.6	Stenosis of pulmonary artery
AS	Q23	Congenital malformations of aortic and mitral valves
	Q23.0	Congenital stenosis of aortic valve
	Q23.1	Congenital insufficiency of aortic valve
	Q23.2	Congenital mitral stenosis
	Q23.3	Congenital mitral insufficiency
	Q24.4	Congenital subaortic stenosis
CoA	Q25.1	Coarctation of aorta
	Q25.2	Atresia of aorta
	Q25.3	Stenosis of aorta
	Q25.4	Other congenital malformations of aorta
TAPVR	Q26.2	Total anomalous pulmonary venous connection
&PAPVR	Q26.3	Partial anomalous pulmonary venous connection
	Q26.4	Anomalous pulmonary venous connection, unspecified
TOF	Q21.3	Tetralogy of Fallot

CHD congenital heart disease, *AVSD* atrioventricular septal defect, *TOF* tetralogy of Fallot, *TGA* transposition of great arteries, *SV* single ventricle, *CoA* coarctation of aorta, *DORV* double outlet right ventricle, *VSD* ventricular septal defect, *PA* pulmonary atresia, *AS* aortic valve stenosis, *PS* pulmonary stenosis, *PDA* patent ductus arteriosus, *ASD* atrial septal defect, *TAPVR&PAPVR* total anomalous pulmonary venous return& partial anomalous pulmonary venous return.

Table S2 The surgical procedures performed for congenital heart disease in patients with and without Down syndrome

Operation	Surgical code[a]	Operation name
Total correction		
	IF180ZN	Total Correction of Tetralogy of Fallot
	IF18101ZN IF18102ZN IF181ZN	Operation Of Ventricular Septal Defect and Pulmonary Valvular Stenosis
	IF1826ZN	RVOT Reconstruction
	IF182AZN	Repair Of Endocardial Cushion Defect-Partial
	IF182BZN	Repair Of Endocardial Cushion Defect-Complete
	IF18502ZN IF185ZN	Repair Of Complicated Congenital Heart Diseases
	IF1861ZN	Left And Right Pulmonary Artery Reconstruction
	IF18701ZN	Rastelli's Operation
	IF18702ZN	Repair Of Total Anomalous Pulmonary Venous Return
	IF18501ZN	Repair Of Transposition of Great Arteries
	IF187BZN	Functional Correction of Single Ventricle-Fontan Operation
	IF1701B	Relief Of Pulmonary Artery Banding
	IF171ZN	Operation Of Atrial Septal Defect
	IF1711ZN	Operation Of Atrial Septal Defect (Minimally Invasive Surgery)
	IF177ZN	Operation Of Atrial Septal Defect and Pulmonary Valvular Stenosis
	IF172ZN	Operation Of Ventricular Septal Defect
	IF167ZN	Operation Of Patent Ductus Arteriosus
Palliation		
	IF1701A	Pulmonary Artery Banding
	IF170A IF17007 IF17006	Shunt Procedure
	IF187AZN	Functional Correction of Single Ventricle-Glenn Operation

[a]The surgical code is based on the international standard operating code of ICD-9-CM (International Classification of Diseases, Ninth Revision, Clinical Modification), which is an international standard. It was selected as a surgical code for each operation.

Table S3 Clinical characteristics of the participants with and without Down syndrome underwent total correction for congenital heart disease

Clinical Characteristics	Observed data (N = 5,282)			Propensity matched data (N = 4.635)		
	Total correction for CHD, N. (%)[a]			Total correction for CHD, N. (%)[a]		
	Control	Down	SMD[b]	Control	Down	SMD[b]
Total, n	5,048	243		4,482	153	
Certain Conditions Originating in the Perinatal Period[c]						
Maternal factors	214 (4.2)	14 (6.0)	0.07	193 (4.3)	8 (5.2)	0.03
Disorders related to length of gestation and fetal	588 (11.6)	44 (18.8)	0.23	511 (11.4)	21 (13.7)	0.05
Birth trauma	56 (1.10)	2 (0.9)	-0.00	51 (1.1)	1 (0.7)	-0.08
Respiratory and cardiovascualr disorder specific to the perinatal period	960 (18.9)	59 (25.2)	0.18	831 (18.5)	32 (20.9)	0.04
Infections specific to the perinatal period	877 (17.3)	57 (24.4)	0.16	794 (17.7)	37 (24.2)	0.15
Haemorrhagic and heamatological disorders of fetus and newborn	1,919 (37.7)	130 (55.6)	0.29	1,767 (39.4)	64 (41.8)	0.04
Transitory endocrine and metabolic disorders	645 (12.7)	31 (13.2)	-0.02	587 (13.1)	21 (13.7)	0.02
Digestive system disorders of fetus and newborn	125 (2.5)	12 (5.1)	0.15	116 (2.6)	3 (2.0)	-0.02
Conditions involving the integument and temperature regulation	166 (3.3)	7 (3.0)	-0.01	154 (3.4)	4 (2.6)	-0.05
Congenital malformations, deformations and other disorders originating in the perinatal period	397 (7.8)	15 (6.4)	-0.05	356 (7.9)	16 (10.5)	0.10
Chromosomal abnormality[d]						
Congenital malformations of the nervous system	135 (2.70)	5 (2.1)	0.02	107 (2.4)	9 (5.9)	0.22
Congenital malformations of eye, ear, face and neck	308 (6.10)	40 (17.1)	0.34	304 (6.8)	18 (11.8)	0.16
Congenital malformations of the circulatory system	5,048 (100.0)	234 (100.0)	-	4,482 (100)	153(100)	-
Congenital malformations of the respiratory system	200 (3.90)	5 (2.1)	-0.16	159 (3.5)	4 (2.6)	-0.07
Cleft lip and cleft palate	67 (1.30)	3 (1.3))	-0.00	52 (1.2)	8 (5.2)	0.35
Congenital malformations of the digestive system	424 (8.30)	24 (10.3)	0.07	385 (8.6)	12 (7.8)	-0.02

Congenital malformations of genital organs	196 (3.90)	12 (5.1)	0.07	176 (3.9)	7 (4.6)	0.02
Congenital malformations of the urinary system	115 (2.30)	8 (3.4)	0.06	101 (2.3)	5 (3.3)	0.07
Congenital malformations and deformations of the musculoskeletal system	409 (8.00)	42 (17.9)	0.29	391 (8.7)	14 (9.2)	0.01
Other congenital malformation	209 (4.10)	5 (2.1)	-0.09	166 (3.7)	4 (2.6)	-0.07

[a]Values are N(%) unless otherwise indicated.

[b]SMD, standardized mean difference. ; a value greater than 10% is interpreted as a meaningful difference.

[c] Certain Conditions Originating in the Perinatal Period is recorded using ICD-10 codes of P00 to P96.

[d]Chromosomal abnormality is recorded using ICD-10 codes of Q00X to Q89.

Table S4 Clinical characteristics of congenital heart disease of the participants with and without Down syndrome underwent total correction for congenital heart disease

CHD	Observed data (N =5,282)			Propensity matched data (N =4.635)		
	Total correction for CHD, N. (%)[a]			Total correction for CHD, N. (%)[a]		
	Control	Down	SMD[b]	Control	Down	SMD[b]
Total, n	5,048	243		4,482	153	
AVSD	585 (11.5)	89 (38.0)	0.63	550 (12.3)	23 (15.0)	0.06
TOF	844 (16.6)	19 (8.1)	-0.21	723 (16.1)	29 (19.0)	0.08
TGA	396 (7.8)	2 (0.9)	-0.39	319 (7.1)	0 (0.0)	-0.38
SV	170 (3.3)	0 (0.0)	-0.24	126 (2.8)	0 (0.0)	-0.23
CoA	346 (6.8)	7 (3.0)	-0.12	294 (6.6)	12 (7.8)	0.04
DORV	113 (2.2)	6 (2.6)	0.00	93 (2.1)	6 (3.9)	0.11
VSD	1,640 (32.3)	91 (38.9)	0.08	1517 (33.8)	59 (38.6)	0.09
PA	43 (0.8)	1 (0.4)	-0.04	39 (0.9)	2 (1.3)	-0.00
AS	27 (0.5)	0 (0.0)	-0.10	22 (0.5)	0 (0.0)	-0.09
PS	112 (2.2)	1 (0.4)	-0.15	102 (2.3)	1 (0.7)	-0.14
PDA	137 (2.7)	5 (2.1)	0.00	119 (2.7)	7 (4.6)	0.11
ASD	568 (11.2)	13 (5.6)	-0.21	525 (11.7)	16 (10.5)	-0.04
TAPVR&PAPVR	46 (0.9)	0 (0.0)	-0.12	36 (0.8)	0 (0.0)	-0.12
OTHERS	21 (0.4)	0 (0.0)	-0.08	16 (0.4)	0 (0.0)	-0.08

[a]Values are N(%) unless otherwise indicated.

[b]SMD, standardized mean difference. ; a value greater than 10% is interpreted as a meaningful difference.

CHD, congenital heart disease; AVSD, atrioventricular septal defect; TOF, tetralogy of Fallot; TGA, transposition of great arteries; SV, single ventricle; CoA, coarctation of aorta; DORV, double outlet right ventricle; VSD, ventricular septal defect; PA, pulmonary atresia; AS, aortic valve stenosis; PS, pulmonary stenosis; PDA, patent ductus arteriosus; ASD, atrial septal defect; TAPVR&PAPVR, total anomalous pulmonary venous return& partial anomalous pulmonary venous return.
